# Chemical risk perception among healthcare workers: formaldehyde exposure as a model for occupational prevention challenges

**DOI:** 10.3389/fpubh.2026.1944564

**Published:** 2026-10-22

**Authors:** Lara García-Asún, Emilio I. Abecia, Begoña Martínez-Jarreta

**Affiliations:** 1 Occupational Medicine Research Group, Aragon-Health Research Institute (GIIS-063), Zaragoza, Spain; 2 Faculty of Medicine, University of Zaragoza, Zaragoza, Spain; 3 Department of Pathology, Miguel Servet University Hospital, Zaragoza, Spain; 4 Institute for Environmental Research (IUCA) of the University of Zaragoza, Zaragoza, Spain

**Keywords:** carcinogen, formaldehyde, healthcare sector, occupational risk, prevention, risk perception

## Abstract

**Purpose:**

Occupational exposure to formaldehyde (FA) in the healthcare sector remains a significant concern due to its carcinogenic and genotoxic potential, particularly among professionals working in pathology and anatomy laboratories. The objective of this study was to analyze the scientific evidence, regulatory frameworks, and chemical risk perceptions associated with formaldehyde (FA) exposure in healthcare settings.

**Methods:**

An integrated methodological design was employed. First, a systematic scoping review of scientific and grey literature was conducted to synthesize health effects and regulatory frameworks regarding FA. Second, an exploratory qualitative descriptive and interpretative study was carried out through two focus groups, comprising physicians and non-physician healthcare professionals, to explore subjective risk perceptions.

**Results:**

The scoping review confirmed formaldehyde’s strong association with nasopharyngeal cancer and low-dose genotoxicity, while findings regarding myeloid leukemia remain inconsistent. Hospital exposure studies revealed that 83.3% of pathology laboratory evaluations documented ambient concentrations exceeding 0.3 ppm, with peak exposures in autopsy rooms reaching up to 8.4 ppm. The qualitative focus groups demonstrated a lower awareness of chemical hazards compared to biological risks, driven by the latent, non-visible nature of chemical exposure. Clear disparities across professional profiles were identified: non-prevention-specialist physicians exhibited lower risk perception and PPE adherence than non-physician healthcare staff. Key operational barriers included inadequate formal training, institutional productivity pressures prioritizing patient flow, and social stigma surrounding safety culture compliance.

**Conclusion:**

These findings underscore the need for optimized preventive strategies, regulatory updates, and targeted awareness campaigns to safeguard worker health. This comprehensive approach highlights the importance of addressing both technical and subjective factors to effectively reduce FA exposure in healthcare settings.

## 1 Introduction

Healthcare personnel are exposed to a broad range of occupational hazards. While biological and psychosocial risks have gained widespread recognition, particularly in the wake of the COVID-19 pandemic, other significant hazards must not be overlooked, among them exposure to chemical substances and their association with serious conditions such as cancer (1).

Cancer is one of the main challenges to public health on a global scale. With an incidence of nearly 20 million new cases and 10 million deaths annually, reducing its impact is an urgent need for the scientific community (2). Of these oncological diseases, it is estimated that between 8% and 10% have an occupational origin (3).

Healthcare professionals working in specific operational settings, such as pathology, anatomy, histology, and laboratory departments, represent a particularly vulnerable occupational subgroup due to routine or high-frequency exposure to hazardous chemical agents (1). One such compound is formaldehyde (FA), classified as a Group 1 carcinogen (carcinogenic to humans) by the International Agency for Research on Cancer (IARC), which is widely used in various professional activities and industries. FA remains one of the most prevalent occupational carcinogens in the European Union, with hundreds of thousands of workers potentially exposed across industrial and healthcare sectors (4). Among occupational sectors, healthcare workers are considered one of the groups at greatest risk of exposure to this substance (5).

FA is a colorless, flammable, and highly reactive gas at room temperature. Due to its chemical properties, it is utilized across more than 50 industrial sectors—including construction, wood and furniture manufacturing, insulation, textiles, paints, and varnishes—as well as serving as a pesticide and antimicrobial agent (6, 7).

Beyond industrial applications, human exposure to FA occurs through both exogenous environmental sources, such as combustion processes, and endogenous biological production via cellular metabolic pathways, the ubiquity of which complicates the determination of strictly occupational exposure origins (8).

In healthcare settings, FA has historically played a fundamental role in hospital pathology departments, forensic medicine, and embalming as a primary fixative for biological tissues (6).

For these clinical applications, FA is commonly used as formalin, an aqueous solution containing approximately 37% FA by weight, typically stabilized with methanol to prevent polymerization (9).

Acute exposure can cause severe caustic damage through ingestion or contact, including coagulative necrosis. Furthermore, as it is gaseous at room temperature, inhalation of FA vapors causes ocular and respiratory tract irritation, as well as nausea. At concentrations exceeding 1.6 ppm, acute exposure can trigger phlegm production, asthma, or laryngospasm, while concentrations above 4 ppm risk inducing severe pulmonary edema (7, 10).

In addition to its acute irritating effects, chronic exposure to FA in workers is a cause for concern because of its potential long-term oncogenic effects. Therefore, its impact on DNA has been investigated. It is known that FA is genotoxic due to its ability to generate covalent bonds with DNA, leading to mutations and clastogenic effects (11).

FA exposure has been associated with nasopharyngeal cancer and certain hematological malignancies, although the strength and consistency of the epidemiological evidence differ by cancer type (12, 66).

It is worth noting that research on the possible impairment of fertility in humans exposed to FA is also underway. A cohort study on the effect of exposure to FA conducted on 205 wood industry workers found that it adversely affected sperm quality (13).

Some studies have also suggested an association between formaldehyde exposure and a greater risk of cognitive decline and amyotrophic lateral sclerosis (ALS) (14).

Given the existing body of knowledge, formaldehyde has been classified by the International Agency for Research on Cancer (IARC) as a Group 1 carcinogen (carcinogenic to humans), with specific reference to nasopharyngeal cancer caused by inhalation (5). Subsequently, in line with recommendations from international agencies, the European Commission classified it as a category 1B carcinogen and category 2 mutagen under European regulations (15).

In the European Union context, the Registration, Evaluation, Authorization, and Restriction of Chemicals (REACH) regulation plays a particularly important role, as its purpose is to improve the protection of human health and the environment from the risks posed by chemicals and chemical mixtures. In support of this regulation, the European Commission published a “Restrictions Roadmap” in 2020, compiling the status of restrictions proposed or under development for various harmful substances (16).

More recently, a historic milestone was reached with the recognition of the right to a safe and healthy working environment as part of what are known as the Fundamental Principles and Rights at Work by the ILO at the 110th International Labour Conference in 2022. Within this framework, it is emphasized that the responsibility for occupational health and safety is shared among various parties, albeit asymmetrically, with the employer always bearing the primary obligation to ensure prevention in the workplace (17).

This initiative aligns with the current Roadmap on Carcinogens 3.0: Strategy 2025–2027, a European initiative aimed at reducing occupational exposure to carcinogenic substances as part of the EU Strategic Framework on Health and Safety at Work (18).

In line with these guidelines, national and international organisations have developed strategies and prevention plans at various levels to improve healthcare worker protection.

Thus, it is also imperative to analyze the implementation of these regulations in real workplace settings, as well as to understand the importance that workers attribute to occupational risks in their workplaces.

The literature indicates that healthcare workers tend to underestimate certain serious health risks, with differences among their professional profiles. Physicians show a lower perception of risk, especially for diseases such as cancer, which may reflect overconfidence or a sense of control and may affect their adherence to preventive measures (19). Various studies have shown that a higher perception of risk translates into a decrease in occupational risk (20–22). Therefore, improving risk perception among professionals and training in occupational risk prevention are particularly important.

While review of the literature enables a systematic mapping of health risks and exposure levels, understanding chemical risk perception within clinical settings requires a contextually grounded, qualitative perspective. The subjective experiences, risk perceptions, and preventive behaviors of healthcare workers regarding formaldehyde remain underexplored. Addressing this knowledge gap is essential for identifying the underlying cultural, organizational, and behavioral barriers that hinder the implementation of effective safety measures in healthcare settings.

Despite the robust regulatory frameworks and documented toxicological evidence surrounding formaldehyde, significant gaps remain regarding how healthcare professionals perceive and manage these risks in daily clinical practice. Existing literature primarily focuses either on technical exposure measurements in industrial settings or on formal regulatory compliance, leaving the subjective risk perceptions, behavioral drivers, and institutional barriers experienced by healthcare workers largely unexplored. Bridging this gap requires an integrated approach that combines a synthesis of current scientific and regulatory evidence with an in-depth qualitative exploration of professional risk perceptions. To address this need, the present study evaluated occupational formaldehyde exposure and prevention in healthcare settings through a dual scoping review and qualitative methodology.

## 2 Objectives

This study sought to explore healthcare professionals’ perceptions of occupational chemical risk associated with formaldehyde exposure, and to analyze how these perceptions influence preventive attitudes and practices within healthcare settings through an integrated scoping review and qualitative approach.

The objectives were as follows.

- To summarize current evidence regarding the health effects of occupational formaldehyde exposure in healthcare settings.
- To analyze current regulatory and preventive frameworks addressing formaldehyde exposure in healthcare environments.
- To explore how healthcare professionals perceive chemical risk and how this perception influences adherence to preventive measures.
- To identify barriers and facilitators affecting occupational safety practices related to formaldehyde exposure.

## 3 Method

In order to achieve the stated objectives, two main approaches were employed: first, a scoping review, which allowed for the synthesis of the available evidence on formaldehyde exposure in healthcare settings; and second, a qualitative study based on focus groups with healthcare professionals, with the aim of exploring the perception of chemical risk associated with formaldehyde in their work environment.

The scoping review framework was structured according to the methodology proposed by Arksey and O’Malley (2005) and updated by the Joanna Briggs Institute (JBI) (23). PRISMA-ScR guidelines (Preferred Reporting Items for Systematic Reviews and Meta-Analyses extension for Scoping Reviews) were applied for structuring and reporting the results.

The objective of the documentary study was defined using the recommended Population, Concept, and Context (PCC) framework as follows:

Population (P): Healthcare workers and hospital personnel (including pathologists, technicians, anatomical prosectors, nurses, and laboratory staff) exposed to formaldehyde in their workplace.

Concept (C): Occupational formaldehyde exposure, associated toxicological and health risks, subjective risk perception, regulatory exposure limits, and preventive/protective measures.

Context (C): Healthcare and hospital settings, specifically pathology departments, clinical and research laboratories, autopsy suites, and anatomy rooms.

In accordance with the recommendations of the PRISMA-ScR framework and the JBI Manual for Evidence Synthesis, differentiated search strategies were adopted for published scientific literature and gray literature, with the aim of capturing the available evidence on the topic as comprehensively and representatively as possible. This combined approach was designed to balance methodological rigor with the flexibility demanded by the scope of the topic, ensuring comprehensive coverage of the available evidence while preserving the systematic integrity of the review.

The systematic academic database search was conducted between October 10, 2024, and November 15, 2024, with a final update search executed on February 15, 2025, to ensure the inclusion of any newly published evidence. The search encompassed four major biomedical databases: PubMed, Scopus, Web of Science, and the Cochrane Library. Gray literature and official documents were also reviewed, including legislation and regulations published in the Boletín Oficial del Estado (BOE) and European Union regulations (EU-OSHA, ECHA, European Commission), as well as documents published by the Instituto Nacional de Seguridad y Salud en el Trabajo (INSST), Spanish Society of Pathological Anatomy (SEAP), Occupational Safety and Health encyclopedia (OSHWiki), National Institute for Occupational Safety and Health (NIOSH), World Health Organization (WHO), and the International Agency for Research on Cancer (IARC).

For the academic databases, search strategies were tailored to each platform’s specific syntax using medical subject headings (MeSH terms) and free-text keywords restricted to title and abstract fields. The exact search string applied in PubMed was as follows:

“Formaldehyde”[Mesh] OR “formaldehyde”[Title/Abstract] OR “formalin”[Title/Abstract]) AND (“Occupational Exposure”[Mesh] OR “occupational”[Title/Abstract] OR “health care workers”[Title/Abstract] OR “pathology workers”[Title/Abstract] OR “laboratory personnel”[Title/Abstract]) AND (“Carcinogens”[Mesh] OR “cancer risk”[Title/Abstract] OR “carcinogenicity”[Title/Abstract] OR “prevention”[Title/Abstract].

Equivalent search strings utilizing database-specific syntax (such as wildcard characters and proximity operators) were executed in Scopus, Web of Science, and the Cochrane Library.

The following criteria were established.

### Inclusion criteria

- Observational studies (cohort, case–control, and cross-sectional), exposure measurement studies, toxicological studies, and previous reviews will be included.
- The study population will comprise healthcare workers exposed to formaldehyde in their workplaces.
- Studies conducted in healthcare settings will be included.
- Articles addressing potential occupational exposure to formaldehyde and providing information on worker risk or protective strategies will be included.
- Articles published in Spanish and English.
- Published from 2017 onwards.
- Articles with available full texts.

### Exclusion criteria

- Studies with a low level of evidence (such as commentaries, letters to the editor, and opinion pieces) will be excluded from the review.
- Research has been conducted exclusively on animals or workers outside the healthcare setting.
- Studies conducted in non-healthcare settings will be excluded.
- Articles with no potential occupational exposure to formaldehyde or those that do not contain information on associated risks or worker protection strategies will be excluded.
- Articles published in languages other than Spanish or English.
- Published before 2017.

The temporal restriction from 2017 onwards was intentionally applied to focus on the most recent evidence regarding healthcare-specific exposure patterns, preventive strategies, biomonitoring approaches, and regulatory developments, while foundational evidence on formaldehyde carcinogenicity had already been extensively established in previous landmark studies and IARC evaluations.

Gray literature and official documents were retrieved through a structured, multi-step process conducted between December 1, 2024, and January 20, 2025. First, targeted manual searches were performed on the official web portals of key regulatory and professional organizations, including the European Commission, EU-OSHA, ECHA, INSST, SEAP, WHO, IARC, NIOSH, and OSHA. On these websites, internal search engines were queried using the terms ‘formaldehyde’, ‘formaldehído’, ‘occupational exposure limits’, and ‘prevention guidelines’. The relevant publications, safety sheets, and technical prevention reports were directly downloaded. To ensure regulatory relevance, documents published within the preceding 30 years were eligible, and the most recent, active versions of regulations and guidelines were systematically retrieved.

To ensure methodological consistency and transparency, we established and applied strict, mutually exclusive definitions for the classification of all retrieved documents:

### Academic literature

Original scientific studies: peer-reviewed journal articles reporting primary empirical data (such as observational, experimental, or cross-sectional studies) specifically focused on formaldehyde exposure, biomonitoring, or risk assessment within healthcare settings.

Peer-reviewed reviews: systematic, scoping, or narrative reviews published in peer-reviewed scientific journals that synthesize evidence on formaldehyde-related health effects, mechanisms, or prevention.

### Grey literature and official documents

Regulatory standards and official reports: authoritative, non-journal publications, technical guidelines, legally binding regulations, consensus statements, or prevention plans issued directly by international, national, or regional governmental bodies (e.g., EU-OSHA, ECHA, INSST, WHO, NIOSH, OSHA) or official scientific and professional societies (e.g., SEAP-IAP) to establish occupational exposure limits or safety protocols.

Foundational peer-reviewed publications prior to 2017 identified during the search were excluded from the systematic review synthesis and instead utilized strictly as supplementary literature within the Introduction and Discussion to provide historical and contextual background.

The study selection process was conducted in three phases:

1. Removal of duplicates using reference management tools.
2. Title and abstract screening was performed independently by two reviewers.
3. Full-text articles were assessed for eligibility.

Any discrepancies arising at either stage were resolved through discussion with a third reviewer until consensus was reached.

The selection process was visually represented using a PRISMA-ScR flow diagram (Supplementary Figure S1). Data from the selected studies and documents were extracted into a structured spreadsheet encompassing the following variables:

- Author (s) and year of publication.
- Country of origin of the study or regulation:
- Type of document (scientific article, review, regulation, or official report).
- The main findings regarding carcinogenicity are as follows.
- Current regulations and applicable legislation are also discussed.
- Strategies and prevention plans for hospitals are discussed.

As recommended in scoping review methodology frameworks, a formal critical appraisal of study quality was not performed, as the aim of the review was to map the breadth and characteristics of the available evidence rather than to provide a quantitative synthesis or establish causal inferences.

Finally, a descriptive analysis was conducted to synthesize the findings and highlight the evidence gaps and current regulations.

A qualitative study within the interpretative paradigm was conducted to examine healthcare workers’ subjective perceptions of the chemical risks associated with occupational FA exposure (24). This type of research was considered appropriate for delving into the subject of study, since the professional lives of healthcare staff are embedded in everyday occupational environments shaped by their respective social and institutional contexts (25).

It is worth noting that this study was conducted as an exploratory qualitative descriptive and interpretative study within an interpretative paradigm. This design is highly suited for exploring the subjective experiences, meanings, and risk perceptions of healthcare workers within their specific everyday clinical and institutional environments (26).

The study was limited to the public and private hospital sectors of the capital city of an Autonomous Community in Spain (Aragón).

Participant selection was based on purposive or judgmental sampling, which allowed for the selection of individuals and locations to describe and understand the central phenomenon of the research. A maximum variation sampling strategy was employed, enabling the capture of diverse perspectives across different professional profiles and work contexts (27). Its use is justified by the complexity associated with the multiple and diverse perspectives of the professionals involved in the phenomenon.

The sampling strategy was implemented through the systematic identification of relevant sampling units, followed by direct approaches to potential informants. Given the demographic composition of the healthcare workforce, the inclusion of both male and female participants was considered essential to ensure representativeness. Subsequent recruitment was facilitated through snowball sampling to identify and engage additional eligible participants.

The characteristics of the 14 participants are presented in Appendix 1.

Data were collected through two focus groups, a conversational technique widely recognized for its capacity to elicit rich accounts of lived experiences and perceptions in specific situational contexts (28).

The groups were constituted according to professional profile: one comprising physicians, and the other, non-physician healthcare professionals. An interview guide was developed and subsequently validated by two experts in occupational medicine. The observation areas and guiding questions are detailed in Appendix 2.

Participants who agreed to take part and met the selection criteria were contacted via email to coordinate the schedule, time, and venue of the session. The interview guide questions were also distributed in advance by email. Anonymity was ensured from the outset, with participants identified by structured codes in which the first two letters indicated professional category, physicians (P) or non-physicians (NP), followed by a letter denoting sex, M (male) or F (female), and the initials of the corresponding specialty. Throughout both sessions, the moderators followed the interview guide, actively encouraged participation, and ensured a balanced and inclusive discussion (28). During the focus group exercise, participants evaluated occupational hazards using a 5-point Likert scale, where 1 represented ‘minimal perceived risk/concern’ and 5 represented ‘maximum perceived risk/concern’.

To ensure the rigor of this qualitative study, several methodological criteria were applied. Credibility (internal validity) was established through triangulation of the focus group data with observational records, with final findings organized into themes. To achieve confirmability (external reliability), Appendix 3 includes all documentary evidence that justifies the process (29).

The study was conducted in accordance with the ethical principles outlined in the Declaration of Helsinki, ensuring participant anonymity and informed consent collection. Ethical approval was granted in April 2025 by the Research Ethics Committee of the Autonomous Community of Aragón (Comité Ético de Investigación de la Comunidad Autónoma de Aragón, CEICA) (Acta No 07/2025). Data processing was registered with the Data Protection Registry of the University of Zaragoza (CUSTOS ID: 100843). Audio recording of the focus group sessions was initiated only after explaining the session’s purpose, guaranteeing anonymity, and obtaining explicit written informed consent from all participants.

Thematic analysis was performed on the focus group transcripts, encompassing both semantic analysis, which describes and interprets the explicit content of participants’ accounts, and latent analysis, which identifies underlying beliefs, assumptions, and institutional norms (30). Thematic recurrence across both focus groups and the absence of substantially novel categories in the later stages of analysis suggested an acceptable level of thematic sufficiency for the exploratory purposes of the study.

The analytical process followed the six-phase framework established by Braun, Clarke, (31) (1) Familiarizing oneself with the data; (2) Generating codes; (3) Searching for themes; (4) Reviewing themes; (5) Defining themes; (6) Writing them up. Phases 2 through 5 were supported by a demonstration version of NVivo qualitative analysis software (32). The analytical orientation was primarily deductive, based on an initial codebook derived from established occupational health frameworks, the CLP regulatory context, and scoping review findings; however, this was enriched inductively by emerging codes arising directly from participant narratives (33).

Data coding and primary thematic analysis were conducted by the first author. The resulting codebook, categories, and thematic structure were subsequently reviewed and verified by a second author to ensure analytical rigor and coding reliability. Discrepancies in code assignment or categorization were discussed and resolved through iterative consensus meetings between the two. Throughout the analytical process, formal researcher reflexivity was maintained: the authors critically evaluated their clinical backgrounds to prevent personal professional assumptions from leading participant narratives or biasing thematic interpretation.

## 4 Results

### 4.1 Scoping review findings

The comprehensive literature search yielded a total of 502 records across databases and secondary sources. In the database search branch, 453 records were retrieved from PubMed, Scopus, Web of Science, and Cochrane Library; after removing 156 duplicates, 297 titles and abstracts were screened, resulting in the exclusion of 184 irrelevant records. Subsequently, 113 full-text reports were assessed for eligibility, of which 97 were excluded for failing to meet pre-established inclusion criteria, yielding 16 eligible peer-reviewed articles. Concurrently, 49 records were identified via official organizations (*n* = 38) and citation searching (*n* = 11); following full-text evaluation of all 49 reports, 35 were excluded (33 non-relevant and 2 not meeting criteria), resulting in 14 official and grey literature documents. Ultimately, a harmonized final sample of 30 documents (16 scientific articles and 14 official/regulatory documents) was included in the scoping review synthesis (Supplementary Figure S1).

These 30 included documents consisted of 18 peer-reviewed scientific publications, comprising 12 original observational and experimental studies (34–45), and 6 systematic/scoping reviews (7, 10, 12, 46–48), and 12 official or regulatory documents representing grey literature, including guidelines and active standards published within the 30-year window (5, 6, 9, 16, 17, 49–55).

Italy accounted for 8 studies (10, 36–39, 42, 43, 47), representing the country with the greatest presence among the evidence sources, followed by Spain with 5 studies (6, 7, 50, 52, 54); Irán, with 5 (34, 35, 40, 45); Kangarlou et al. (53) and the United States (US) with 4 (9, 49, 53, 55). Two documents were international in scope (5, 17) and two originated from the European Union (16, 51). The following countries contributed with one study each: France (12), South Korea (46), Malasya (48) and China (44). Of these, 3 documents were published between 2006 and 2017 (5, 50, 54), 6 between 2018 and 2020 (7, 12, 43, 45, 46, 49) and 21 between 2021 and 2024 (6, 9, 10, 16, 17, 34–42, 44, 47, 48, 51–53, 55). The characteristics of the documents found are summarized in the results table in the appendices (Appendix 4). An example of the table is shown, presenting the data extracted from the first three studies (Table 1).

**Table 1:** Data extraction table example.

Reference	Year	Document type	Country/region	Population	Source	Conclussions
Kwon et al. (46)	2018	Systematic review	South Korea	54,000 laboratory workers in anatomical pathology, endoscopy, and furniture manufacturing	Database	This study showed a significant association between formaldehyde exposure and the development of leukaemia (especially myeloid) and Hodgkin’s lymphoma, with higher risks in peak exposures ≥4 ppm. However, the relationship with nasopharyngeal cancer (NPC) is less clear, showing significant results in case–control studies but not in cohorts, suggesting the influence of environmental factors or biases on the results of these studies. Although South Korea recognized formaldehyde as an occupational carcinogen in 2013, there have been no cases compensated solely for this exposure.
Ghelli et al. (37)	2021	Case–control study	Italy	Exposed group: 23 hospital workers (nursing staff, anatomical pathology technicians, and pathologists). Control group: 45 hospital workers without exposure.	Database	Workers exposed to formaldehyde showed higher levels of oxidative stress (15-F_2 t_-isoprostane and MDA) and inflammation (TNF-α) than those of unexposed workers (*p* < 0.001). A positive correlation was found between airborne formaldehyde concentration and oxidative stress biomarkers (MDA: rho = 0.647, *p* < 0.001). Subjects with GSTT1 and GSTM1 genotypes and those carrying mutated alleles in CYP1A1 showed higher levels of MDA (*p* = 0.038, 0.031, and 0.008, respectively), suggesting a greater vulnerability to oxidative damage.
Ghelli et al. (38)	2022	Case–control study	Italy	Exposed group: 57 pathologists. Control group: 48 healthcare workers without exposure.	Database	Pathologists exposed to formaldehyde displayed a higher frequency of sister chromatid exchanges (SCEs) in blood lymphocytes, supporting its genotoxic effect, even at low concentrations. Airborne formaldehyde concentration was directly correlated with SCEs and inversely correlated with the replication index (RI), indicating its cytotoxicity. Although some polymorphisms (CYP1A1 exon 7 and XPC exon 9) were associated with a higher frequency of SCEs in the exposed group, regression models did not show a significant role for the analysed genetic variants in modulating genotoxicity.

The following is a synthesis of the main findings of this study. The results are organized into three main categories for the sake of clarity.

#### 4.1.1 Scientific evidence on the chronic effects of formaldehyde exposure in healthcare settings

Seven studies analyzing the chronic effects of occupational FA exposure among healthcare workers were reviewed.

Evidence compiled by the IARC (5) and Kwon et al. (46) demonstrates site-specific oncological risks associated with occupational formaldehyde exposure. The IARC confirmed a causal link with nasopharyngeal carcinoma and highlighted elevated risks for leukemia, particularly myeloid leukemia, among exposed professionals, supported by case–control studies in embalmers and funeral workers. These epidemiological observations are biologically supported by network analyses showing that formaldehyde induces reactive oxygen species, oxidative stress, and apoptotic dysregulation in hematopoietic stem cells (46). Finally, reported associations with sinonasal cancer remain subject to potential residual confounding from co-exposure to wood dust (5).

Biologically, exposure is associated with oxidative stress and genotoxicity, as evidenced by chromosomal alterations and other biomarkers (34, 37–39).

Jalali et al. (40) reported that, in the pathology laboratories under study, the mean carcinogenic risk attributable to formaldehyde exposure (3.45 × 10^−4^) exceeded acceptable thresholds according to occupational health standards in 81.7% of the subjects assessed.

#### 4.1.2 International, European, and Spanish regulatory frameworks related to occupational exposure to formaldehyde

Eight documents lay the strategic and legislative foundations for protecting workers against occupational risks such as FA across international, European, and national jurisdictions.

In 2021, the EC presented a strategic framework for occupational health and safety (2021–2027), prioritizing the prevention of occupational diseases, particularly occupational cancer, and proposing updates to existing legislation and enhanced protection for vulnerable groups, including healthcare personnel (51). Additionally, in collaboration with the ECHA, FA has furthermore been included in prioritization lists for restriction on the grounds of occupational risk in healthcare settings, where current exposure limits may prove insufficient (16). In the United States (USA), OSHA establishes specific FA exposure standards, encompassing permissible limits, environmental controls, personal protective equipment requirements, and medical surveillance protocols (9). In Spain, the INSST annually sets occupational exposure limits, incorporating FA since 2018 (52). National legislation further recognizes diseases resulting from FA exposure as occupational diseases of chemical origin, provided that a causal relationship is demonstrated (54).

Loomis et al. (12) updated the list of occupational carcinogens identified by the IARC between 1971 and 2017 using strict criteria to ensure relevance and causality, among which FA was included.

Internationally, the 110th International Labour Conference (ILC) provides a reference framework for regulatory compliance, underscoring the need to align national legislation with international standards and emphasizing the importance of implementing sector-specific preventive measures in high-risk settings, such as healthcare (17).

#### 4.1.3 Prevention and protection strategies for workers exposed to formaldehyde

17 articles referenced the identification of risk situations, as well as plans and strategies for the prevention of occupational diseases.

This review included six articles demonstrating FA exposure levels in hospital settings (7, 35, 36, 41, 42, 45). When reporting exposure levels across the included studies, concentrations were categorized by sampling metric to ensure valid comparisons. Ambient formaldehyde levels exceeded the European 8-h Time-Weighted Average (TWA) limit of 0.3 ppm in 5 out of 6 studies during routine laboratory tasks (7, 35, 36, 41, 45). Peak exposures exceeding short-term limits (STEL), reaching up to 8.40 ppm, were specifically documented during high-emission procedures such as organ trimming, embalming, and autopsies (7, 41). Only Motta et al. (42) in their longitudinal study, found concentrations consistently below 0.16 ppm. Regarding carcinogenic risk, Yahyaei et al. (45) and Foroughi et al. (35) showed levels above the safe thresholds, whereas Kangarlou et al. (41) showed these thresholds were not exceeded.

Some studies have also verified the effectiveness of certain preventive measures. Measures recognized as effective in reducing occupational FA exposure in anatomical pathology laboratories include spatial reorganisation and forced ventilation (36, 43), reducing the amount and frequency of formaldehyde use (36, 44), the use of personal protective equipment (PPE) (43, 44) and worker education and awareness of occupational risks (36, 42, 44).

Substituting formaldehyde with another substance that has similar fixation and staining properties remains an ideal goal. Different substances have been evaluated to date, but no consistent results have been obtained (48, 50).

Biomarkers and monitoring strategies for FA exposure were also evaluated. Real-time continuous monitoring improves the detection of short-term exposure peaks compared with traditional time-integrated sampling methods. Regarding biological monitoring, traditional exposure biomarkers, such as urinary formic acid (main metabolite of absorbed formaldehyde), present notable diagnostic limitations due to background endogenous production and low analytical specificity. In contrast, cytogenetic assays (such as micronucleus tests and sister chromatid exchanges) serve as biomarkers of early biological or genotoxic effect rather than specific exposure biomarkers for formaldehyde. These early-effect biomarkers demonstrate higher sensitivity in detecting early DNA damage and could be valuable tools for occupational health surveillance, although proper adjustment for confounding factors is required (7, 47).

The Spanish Society of Pathology (SEAP-IAP) (50) NIOSH (49, 53), Environmental Health and Safety Office (EHS) of Yale University (55) and the INSST (6) have developed good practice guidelines to reduce occupational risk from formaldehyde exposure, including information about its uses, monitoring, environmental limits, preventive measures, use of protective equipment, and actions in case of poisoning. Notably, recommendations for specific measures and possible reassignment of pregnant or breastfeeding workers due to reproductive risks have been made (53).

Two reviews highlighted the importance of an integrated “One Health” approach (“One Health”). Charlier et al. (10) emphasize the importance of measures that reduce formaldehyde exposure in a global way.

### 4.2 Qualitative focus group findings

The results of the thematic analysis of the focus groups revealed a set of experiences, perceptions, and meanings shared by the participants regarding their perceptions of formaldehyde risk. Through coding and refinement, five key themes were identified that captured the core dimensions of the phenomenon under study (Table 2). These themes reflect both consensus and discursive differences within the groups, offering a nuanced understanding of the situations experienced by the professionals.

**Table 2:** Identified themes and subthemes from qualitative focus group analysis.

Theme 1: FA exposure and risk assessment	Theme 2: risk management and preventive measures	Theme 3: factors influencing adherence to measures	Theme 4: chemical risk vs. other occupational risks	Theme 5: occupational risk prevention in legislation
- Subtheme 1.1. Risk situations and environmental limits - Subtheme 1.2. Biomonitoring and biological limit values - Subtheme 1.3. Difficulties in risk assessment: causality and individual susceptibility	- Subtheme 2.1. Individual measures - Subtheme 2.2. Collective measures - Subtheme 2.3. Evolution of measures	- Subtheme 3.1. Hindering factors - Subtheme 3.2. Enabling factors	- Subtheme 4.1. Beliefs - Subtheme 4.2. Differentiated risk management	- Subtheme 5.1. Legislative development - Subtheme 5.2. Obligations of companies - Subtheme 5.3. Obligations of workers

Thematic matrix categorizing qualitative findings across five core domains: risk assessment, preventive measures, factors influencing adherence, comparative risk perceptions, and regulatory compliance among healthcare professionals.

#### 4.2.1 Exposure to FA and risk assessment

##### 4.2.1.1 Risk situations and environmental limits

Regarding formaldehyde exposure, qualitative accounts from dissection technicians and anatomical pathologists reflected perceived discrepancies between routine workplace conditions and occupational exposure limits (OELs). Participants described subjective experiences indicating that environmental and personal monitoring during high-risk procedures, such as embalming and fresh autopsies, routinely yielded values exceeding established legal limits. In this context, one dissection technician emphasized the frequent exceedance of exposure limits during personal sampling, stating:

*“The problem is that personal monitoring during certain procedures always comes out over the limit [referring to values repeatedly exceeding occupational exposure thresholds]. They always come out high”.*

These experiences are entirely consistent with those documented in other European contexts, reflecting a widespread and recurring pattern of difficulty in meeting the OELs currently in force in the EU.

Furthermore, participants emphasized that air sampling was conducted only sporadically, leaving clinicians uncertain about actual concentration levels during prolonged autopsy procedures.

*“You are performing a clinical autopsy — four, five, six hours — breathing in that environment, and of course, the FA levels… who knows how high they are. No measurements are taken there”.*

##### 4.2.1.2 Biomonitoring and biological limit values

The focus groups revealed widespread skepticism toward the usefulness of urinary formic acid as a biomarker for FA exposure. Participants noted that, although formic acid can be measured, no established biological limit values (BLVs) exist to allow the results to be interpreted clinically or in a preventive context:

*“The issue is that there are no biological limit values. They can test for formic acid, but we have nothing to compare it to because there is no limit value telling us, ‘from here on’”.*

Notably, participants did not spontaneously mention advanced biomonitoring strategies, such as cytogenetic assays.

##### 4.2.1.3 Challenges in risk assessment

Nurses and occupational physicians identified two main difficulties in assessing FA-related risks: challenges in establishing causality and the role of individual susceptibility. One oncology nurse highlighted the lack of routine occupational history-taking in general clinical practice as a barrier to identifying potential work-related etiologies, citing a patient’s observation during routine care:

*“The other day a patient told me: ‘You ask a lot of questions,’ she said, ‘but you never ask what we do for work’”.*

This statement reflects general clinical history-taking practices rather than a specific documented exposure to formaldehyde.

#### 4.2.2 Risk management and preventive measures

##### 4.2.2.1 Individual measures

A clear divergence emerged between professional categories regarding the selection and personal use of personal protective equipment (PPE). Non-physician healthcare staff (technicians and nurses) expressed higher risk concern, proactively demanding appropriate respiratory protection (such as filtering facepiece respirators with organic vapor cartridges). Conversely, physicians acknowledged the hazard but reported inconsistent Respiratory Protective Equipment (RPE) use. A pathology physician acknowledged knowing the risks while admitting to omitting respiratory protection during routine tasks.

##### 4.2.2.2 Collective measures

There was unanimous agreement that substituting formaldehyde with safer alternatives would represent the ideal solution. However, technical staff described operational barriers experienced during local trial phases with commercial non-formaldehyde fixatives. They reported that these attempts were discontinued due to adverse health reactions among personnel, specifically contact dermatitis and mucosal irritation, alongside concerns regarding diagnostic preservation quality.

*“They have tried everything; they even tried using an alternative commercial substitute, but it triggered skin reactions and eye irritation in the staff handling the samples”.*

Authors explicitly clarify that this single localized trial reflects context-specific operational barriers and worker perceptions, rather than evidence that global formaldehyde substitution is technically unfeasible.

Participants also shared personal perceptions regarding workplace ventilation, expressing concern that ceiling-mounted extraction systems might draw vapors upward through their breathing zone. Authors clarify that these accounts reflect workers’ subjective perceptions of localized airflow rather than technical engineering evaluations of ventilation design. The occupational hygiene literature demonstrates the effectiveness of spatial reorganization and engineering controls such as replacing overhead canopy hoods with localized backdraft or downdraft local exhaust ventilation (LEV) systems positioned at table height, which reduce breathing-zone concentrations (36, 43).

*“When you think about extraction, you place it on the ceiling, but if you place it on the ceiling it passes through your breathing zone before it leaves”.*

Participants recognized positive historical advancements in protective equipment availability, noting improvements in equipment ergonomics and gender-adapted sizing. A female staff member noted:

*“Considerable progress has been made, and we now have personal protective equipment specifically sized for female personnel”.*

#### 4.2.3 Factors influencing adherence to preventive measures

##### 4.2.3.1 Hindering factors

Risk perception emerged as an important factor influencing safety behaviors, with greater awareness generally associated with greater self-reported adherence to preventive measures. As one participant explained:

*“What happens in pathology services is that in the end, when you use something every day you become complacent, you lose your fear”.*

The prioritization of patient flow and clinical efficiency over personal protection also emerged as key barrier, particularly among physicians, who reported viewing PPE as an impediment to care delivery.

*“I would invariably put on a surgical mask when attending to an oncology patient undergoing chemotherapy, to protect the patient. (…) But it never occurred to me to wear it for my own protection (…). It wasn’t due to external pressure; it was just how things worked, because I didn’t perceive the risk for myself”.*

These findings should be interpreted as exploratory and context-dependent observations derived from the qualitative component of the study, rather than as universally generalizable conclusions about professional cultures or behaviors.

Deficiencies in formal training were highlighted across all groups, with institutional management assuming safety skills were acquired during undergraduate education:

*“At the places where I have worked, risk prevention (…) it’s taken for granted that you already know everything”.*

Furthermore, non-physician staff reported encountering peer resistance or negative social reactions when adhering strictly to protective protocols. *“The truth is that if you take this issue seriously, they start to give you funny looks”.*

*“I’ve been mocked many times because I kept wearing a surgical mask after it was no longer required”.*

Physicians appeared to perceive this social pressure to a lesser extent:

*“When there is an adequate perception of the risk, there is no mocking at all”.*

##### 4.2.3.2 Enabling factors

The study reveals two distinct approaches to promoting PPE use among professionals. Among nurses and technicians, a practice-led model predominates, emphasizing the importance of supervisors and trainers consistently modeling safe behaviors:

*“If your supervisors wear them, you’ll have to wear them too. I mean, I can be very aware and they always see me with the RPE on”.*

In contrast, physicians emphasized the need for clear consequences and progressive sanctions to ensure compliance, citing cases in which firm disciplinary measures led to behavioral change.

#### 4.2.4 Perception of chemical risk versus other occupational risks

##### 4.2.4.1 Beliefs

To complement the qualitative findings, participants evaluated five major categories of occupational hazards using a 5-point scale (where 1 indicated the lowest perceived risk and 5 the highest). Aggregating the rating scores across all participants (*n* = 16) established a clear hierarchy of perceived workplace hazards. Occupational risks were prioritized from highest to lowest concern as follows: (1) Biological risks, (2) Physical/radiation risks, (3) Psychosocial risks, (4) Chemical risks, and (5) Ergonomic risks. Notably, chemical hazards were ranked fourth overall, demonstrating that healthcare workers subjectively perceive chemical risks as less immediate or hazardous compared to biological or physical threats.

This disparity was attributed to the immediate, visible consequences of biological injuries and well-known contagious diseases’ risks compared to the latent, non-visible nature of chemical exposures:

*“I think we underestimate the risks we don’t see. If I contract HIV, I know the consequences and I know exactly the risk I’m taking. Formaldehyde is something ethereal; you don’t know whether you’ll be affected or not”.*

##### 4.2.4.2 Management

Similarly, participants noted that training has traditionally emphasized protocols for biological risks, and more recently, psychosocial risks, while chemical risks have received comparatively less attention. They reported this disparity is reinforced by cultural factors, such as media representations of certain hazards and the recent COVID-19 pandemic, which have contributed to normalizing certain protective behaviors over others:

*“You see the radiation hazard sign. We’ve all seen movies about nuclear disasters, and that sticks with people more deeply”.*

#### 4.2.5 Occupational risk prevention in legislation

##### 4.2.5.1 Legislative development

Occupational health professionals acknowledged regulatory progress following the European Union’s reclassification of FA as a Category 1B carcinogen and Category 2 mutagen, which they perceived has driven important improvements since approximately 2015.

##### 4.2.5.2 Responsibilities of employers

Occupational safety experts highlighted the legal requirements imposed on organizations, under penalty, including an initial risk assessment as well as periodic reassessments, health surveillance of workers, and specific training programs:

*“It is mandatory for each company to indicate the risks associated with each job position for each worker, and to inform and train them”.*

However, testimonies from occupational health nurses suggest opportunities to optimize the practical application of these measures. While organizations may have well-structured protocols, their operational deployment shows disparities depending on the specific work context.

A particular concern was raised regarding institutional protocols protecting pregnant or breastfeeding workers. Testimonies revealed that fear of professional repercussions led workers to delay pregnancy disclosure during critical first-trimester periods, and although institutions have relocation protocols, their application is not always immediate or uniform in practice due to resource constraints:

*“You hesitate to disclose your pregnancy, and you’re working with the fear of, ‘wow, the first month or the first two months when a baby is developing are so important, will it affect me or not?’, but you don’t say anything. Out of fear. What will they do with me?”*

##### 4.2.5.3 Responsibilities of workers

The group of physicians Participants agreed that workers bear a legal and ethical duty to comply with established safety measures, noting that peer accountability reinforces compliance.

*“A worker who does not want to use (the PPE) cannot be working, because it’s a risk for him, for the company itself, and for everyone. They are risks, we’re talking about risk”.*

## 5 Discussion

### 5.1 Formaldehyde exposure, carcinogenicity, and regulatory frameworks

To ensure methodological transparency, the evidence presented in this section is categorized into two distinct sources: (1) primary findings derived directly from the documents included in the scoping review and the qualitative focus groups, and (2) supplementary scientific literature introduced within the Discussion to contextualize, contrast, or ground the findings in broader epidemiological and theoretical frameworks.

The scoping review is consistent with the previous evidence that occupational exposure to FA among healthcare professionals is associated with an increased risk of cancer, supported by the epidemiological evidence compiled in IARC Monograph 100F (5). While the association with nasopharyngeal cancer is widely accepted in the scientific literature, heterogeneity exists between case–control studies, which report a significant association, and cohort studies, which yield inconsistent results—suggesting the influence of confounding factors or methodological biases (56–58). The link with leukemia remains more controversial. Despite the biological plausibility for its development (59), supported by studies from (15, 37, 38), the epidemiological evidence is limited, and recent reviews have questioned direct causality due to confounding factors and methodological limitations (8, 60).

Regarding other categories of risk discussed in the literature, such as effects on fertility (13) and the nervous system (14, 61, 62), no studies specifically focused on healthcare professionals were identified in the search.

It should be noted that most higher-level evidence studies, such as cohort studies and those employing biomarkers, originate from industrial settings or are not exclusively focused on healthcare contexts. In the latter, a lack of robust, large-sample studies is observed, which would be necessary to yield more meaningful results. The use of co-exposures such as xylene or anesthetic gases represents an additional confounding challenge (10). Further complexity arises from the rapid introduction of new workplace substances, which outpaces the capacity to assess their health effects (12).

The findings of this review highlight significant limitations in the current literature. There is a clear disparity between the abundant evidence from industrial studies and the scarcity of robust data specifically designed for healthcare settings. This gap is particularly relevant, given that exposure patterns in hospitals and medical laboratories, involving intermittent exposure at both low and high doses, differ substantially from those in industrial contexts, where exposure tends to be continuous and at high concentrations.

Another critical gap is the near-total absence of research on other possible systemic effects of occupational FA exposure in healthcare professionals, particularly regarding reproductive function, pregnancy, breastfeeding, and neurological health. This knowledge gap is especially concerning given the high proportion of individuals of childbearing age employed in this sector.

To address these limitations, the following research priorities are recommended: prospective multicenter cohort studies specifically designed for healthcare populations, with long-term follow-up and periodic monitoring of individual exposure levels through personal dosimetry, early-effect biomarkers, and clinical outcomes; and systematic evaluation of non-neoplastic effects, particularly those related to pregnancy and reproductive function, as well as markers of neurotoxicity and cognitive impairment.

At the regulatory level, analysis of the identified documents reveals a framework that, while demonstrating significant progress over the past decade, remains heterogeneous in its application and scope.

Notably, considerable differences are observed in the occupational exposure limits (OELs) established by different jurisdictions, as shown in Table 3.

**Table 3:** Permissible exposure limits (PELs) for FA across regulatory entities.

Entity	Last update	30 min EL*	TWA**	STEL***
OSHA (9)	2021	—	0,75 ppm	2 ppm
OMS (11)	2010	0,08 ppm	—	—
INSST (1)	2024	—	0,3 ppm	0,6 ppm

*30-min exposure limit for general indoor air quality **time weighted average, ***short-term exposure limit.

These divergences reflect not only differing interpretations of the available scientific evidence, but also different approaches to the precautionary principle, particularly relevant in the context of chronic low-dose exposures, which are characteristic of healthcare settings.

It is also notable that the established limits have changed little over the past two decades, which may suggest that they remain partly anchored in the prevention of acute effects and do not adequately incorporate emerging evidence on carcinogenicity, particularly the genotoxic potential of FA at low doses demonstrated in the reviewed literature (34, 37–39).

Analysis of current norms and regulations reveals disparities in exposure limits between agencies, regulatory rigidity in the face of recent evidence, and difficulty establishing causal links in multifactorial environments, all of which limit their practical effectiveness. These findings underscore a clear need to evolve toward a more robust and up-to-date regulatory framework, along with a comprehensive review of worker protection policies. This could be achieved through periodic reviews of exposure limits incorporating the latest findings on genotoxic and carcinogenic effects at low concentrations, the development of sector-specific regulations that account for the particularities of each workplace, and the promotion of uniform implementation of available preventive measures. Although formaldehyde remains classified as a Group 1 human carcinogen, the IARC Advisory Group has recommended it as a high-priority agent for re-evaluation during the 2025–2029 period, considering it ready for evaluation within this five-year window (5).

The results of this scoping review also highlight a concerning situation regarding formaldehyde exposure in hospital settings, particularly in pathology laboratories. The data show that in 5 out of the 6 analyzed studies (83.3%), environmental concentrations exceeded the European 8-h Time-Weighted Average (TWA) limit of 0.3 ppm (7, 35, 36, 41, 45). Though given the small number of studies evaluating ambient exposure this proportion should be interpreted with caution, these findings align with those indicating carcinogenic risk above safe thresholds across various studies (35, 45), suggesting that healthcare staff, particularly those in pathology departments, constitute a particularly vulnerable group.

The preventive measures assessed show varying degrees of effectiveness. Spatial reorganization, forced ventilation, the use of closed extraction hoods (36, 43), reduction of FA use, PPE provision, and worker training and awareness programs appear to be the most effective interventions, yielding significant reductions in environmental concentrations.

The measures evaluated that have demonstrated a reduction in FA exposure, along with their level of evidence and implementation challenges, are presented in Table 4.

**Table 4:** Overview of occupational preventive strategies, level of evidence, and implementation challenges.

Strategy	Evidence	Implementation Challenges
Substitution (48, 50)	Effectiveness in reducing exposure is not directly assessed; however, complete substitution is assumed to eliminate risk by removing FA exposure entirely	Lack of validation in routine diagnostics
Ventilation and spatial reorganisation (36, 43)	Analytical observational study	High cost
PPE (43, 44)	Analytical observational study	Lack of awareness
Use reduction (36, 44)	Analytical observational study	Lack of standardized protocols
Training and awareness (36, 42, 44)	Analytical observational study	Lack of standardized protocols

Evaluation of main occupational control measures (chemical substitution, localized exhaust ventilation, personal protective equipment, exposure reduction, and worker training) synthesized from the scoping review.

Additional measures showing promise for improving health surveillance include real-time automated sampling systems (43) and early-effect biomarkers (7, 47).

These findings underscore the need to strengthen the protection of healthcare personnel against FA, prioritizing the development of safer alternatives that maintain diagnostic quality. It is equally important to improve surveillance through continuous environmental monitoring, use of genetic damage biomarkers, and clinical-occupational records, enabling comprehensive follow-up. These actions should be integrated into strategies combining environmental control, training, and targeted measures for vulnerable groups, whose effectiveness should be evaluated through longitudinal studies. All of this requires close collaboration among healthcare, scientific, and regulatory stakeholders to advance the reduction of FA-associated risks.

The following section presents the results of the qualitative study, contrasting the perspectives and experiences of the various professional profiles involved. These findings are further compared with the evidence gathered in the scoping review and the theoretical framework, enabling an assessment of concordance or divergence between the literature and the reality as perceived by professionals.

### 5.2 Interpretation of qualitative insights: behavioral factors, safety culture, and compliance discrepancies

The discussion below is organized according to the five core thematic domains and thirteen subthemes identified in the study, contrasting qualitative accounts between professional groups and evaluating their concordance with quantitative literature and regulatory frameworks.

#### 5.2.1 Exposure to FA and risk assessment

##### 5.2.1.1 Risk situations and environmental limits

The qualitative reports regarding perceived elevated FA levels during high-risk procedures interpretively mirror the empirical measurement literature, which documents that 83.3% of hospital pathology laboratory evaluations exceed the EU 8-h Time-Weighted Average limit of 0.3 ppm, reaching short-term peak exposures up to 8.40 ppm during autopsy procedures (7, 36, 41). However, while published quantitative studies evaluate controlled laboratory environments, the qualitative findings reveal a potential blind spot in clinical practice: the reported total absence of exposure monitoring in some clinical autopsy suites, where personnel undergo unmonitored exposures during prolonged sessions.

##### 5.2.1.2 Biomonitoring and biological limit values

Staff skepticism regarding urinary formic acid diagnostic utility aligns with established toxicological evidence: it exhibits low diagnostic specificity and sensitivity as an exposure biomarker due to background endogenous metabolic production and dietary confounders (7, 47). This limitation is compounded by the absence of established biological limit values (BLVs), which participants agree creates an interpretive void for occupational health services and hinders clinical surveillance (1).

Although cytogenetic assays were not spontaneously cited by focus group participants, this finding must be interpreted with caution. Because participants were not explicitly questioned about cytogenetic testing, their omission highlights the operational gap in translating advanced genotoxic research into health surveillance protocols, rather than a lack of professional awareness.

##### 5.2.1.3 Challenges in risk assessment

While toxicological and epidemiological literature identifies multi-chemical exposures, long latency periods, and individual genetic susceptibility as primary factors complicating causal attribution for chronic pathologies (10), the qualitative findings reflect healthcare workers’ personal perceptions regarding these diagnostic hurdles. Specifically, participants reported a shared perception that comprehensive occupational medical histories are often omitted during clinical consultations, describing this as a barrier to the long-term health surveillance recommended in preventative literature (63).

#### 5.2.2 Risk management and preventive measures

##### 5.2.2.1 Individual measures

The qualitative study reveals that adherence to protective measures against formaldehyde varies among healthcare professionals, with a notable gap observed between physicians and non-physicians. Non-physician personnel demonstrated higher risk concern and active demands for appropriate measures. This contrast in PPE use is consistent with the literature, which indicates that physicians tend to have a lower perception of occupational risk for serious conditions, including cancer, compared to other healthcare professionals, placing greater emphasis on maintaining workflow efficiency (19, 21).

##### 5.2.2.2 Collective measures

While chemical substitution is the highest priority in occupational hygiene, focus group accounts reflected a widespread perception among staff that replacing formaldehyde remains impractical. Participants described trial failures with alternative fixatives. Although these accounts represent individual experiences rather than technical proof, they align with literature showing that alternative fixatives currently fail to match formalin in diagnostic reliability (48, 50).

Participants perceived that overhead systems draw vapors upward across their breathing zone. This qualitative perception aligns with airflow dynamics: overhead hoods can draw volatile vapors through the breathing zone, whereas backdraft or downdraft LEV tables capture vapors at the source, pulling them away from the operator (36, 43). Finally, workers recognized the utility of airtight formalin containers during transport, but identified an operational limitation: opening them during specimen manipulation creates residual exposure peaks requiring secondary controls.

There was consensus on the positive progress made regarding preventive measures and their accessibility regardless of body type or gender, reflecting institutional commitments to equitable occupational safety equipment design (17, 51).

#### 5.2.3 Factors influencing adherence to preventive measures

##### 5.2.3.1 Hindering factors

Focus group accounts illustrated how daily, routine handling of formalin leads to risk normalization and habituation, and how higher risk awareness generally correlates with increased self-reported adherence to safety protocols, these phenomena being well documented in literature (20, 21).

The perceived tension between productivity demands and personal protection further highlights a documented difference in priorities. While nurses and technicians reported experiencing operational workload pressures, their qualitative accounts emphasized a strong commitment to self-protection. On the other hand, the reported tendency among some physicians to prioritize clinical workflow over personal protection reflects professional identity norms where patient care takes precedence over personal risks (64). These findings should be interpreted as exploratory and context-dependent observations derived from the qualitative component of the study, rather than as universally generalizable conclusions about professional cultures or behaviors.

##### 5.2.3.2 Enabling factors

The distinct strategies proposed by participants to enhance compliance reflect differences in professional teaching cultures. Non-physician personnel favored a leadership model. In contrast, physicians advocated for formal administrative enforcement and progressive sanctions. These findings might indicate that these approaches are complementary rather than mutually exclusive.

#### 5.2.4 Perception of chemical risk versus other occupational risks

##### 5.2.4.1 Beliefs

The analysis reveals that risk perception varies substantially according to risk type, with greater awareness of biological compared to chemical risks among healthcare workers in both groups, the latter being perceived at a level comparable to psychological risks. Participants attributed this difference to several factors, including the immediacy and visibility of consequences in biological risks versus the latent and less tangible nature of chemical hazards.

The higher risk perception observed for biological and radiological hazards compared to chemical hazards is interpretively explained by health belief models: biological injuries offer immediate culturally dramatized consequences, whereas chemical carcinogenesis presents a latent, intangible risk profile (19, 22). Because human cognition naturally underestimates delayed health outcomes, relying on voluntary personal compliance is inherently insufficient for chemical safety. Findings suggest that to bridge this gap, institutions must focus on two structural interventions: making invisible chemical hazards tangible through continuous, real-time environmental monitoring systems with visual feedback, and prioritizing automated engineering controls that passively protect personnel regardless of individual risk perception or workload pressures (20, 43).

##### 5.2.4.2 Management

Institutional training and hospital protocols remain heavily biased toward infection control and radiation safety, leaving chemical prevention under-resourced (Charlier et al. (15)). The combination of these factors may explain why among participants measures such as glove use or radiological protection are more firmly established, while those relating to chemical risks show lower adherence.

#### 5.2.5 Occupational risk prevention in legislation

##### 5.2.5.1 Legislative development

Perception of regulatory development among participants is consistent with the regulatory framework outlined in the scoping review, notably the EU reclassification of formaldehyde as a Category 1B carcinogen and Category 2 mutagen, which has driven updated national occupational exposure limits since 2015–2018 (1, 51, 54).

##### 5.2.5.2 Responsibilities of employers

While participants recognized statutory employer requirements regarding initial risk assessment and periodic reassessment, health surveillance, and training (1), qualitative accounts revealed critical operational barriers regarding vulnerable workers. Pregnant personnel faced anxiety and administrative delays during early pregnancy, leading to potential exposure during sensitive first-trimester developmental windows. This suggests an operational gap between formal regulatory protections (53) and their real-world implementation in hospital settings.

##### 5.2.5.3 Responsibilities of workers

Participants recognized that workers hold a statutory and ethical duty to comply with established safety measures, noting that positive peer accountability serves as an effective mechanism to reinforce compliance within clinical teams.

The study of chemical risk perceptions among healthcare professionals remains an underexplored area. There is an acknowledged need for research integrating qualitative methodologies (64). The present study contributes qualitative evidence that deepens the understanding of the current situation from the perspective of healthcare workers. Future research should address aspects that have received limited attention to date, particularly the need to promote attitudinal change among certain professional groups regarding the perception of chemical risks. While training, information, and awareness campaigns constitute valuable tools, fostering genuine attitudinal change among those with lower risk awareness remains of paramount importance. Some studies already highlight the significance of the information source, message, and medium in achieving persuasive impact (65). Evaluating the effectiveness of such campaigns in changing attitudes and fostering a culture of prevention represents one of the key challenges in this field.

## 6 Limitations

This study presents several methodological limitations that should be considered when interpreting the findings, structured around its two main components.

Regarding the scoping review, search criteria were restricted to publications in English and Spanish, which may have excluded relevant evidence published in other languages. Additionally, eligibility criteria limited scientific literature to publications from 2017 onwards to map the most recent healthcare-specific evidence and preventive frameworks; earlier empirical studies were therefore not systematically included, although landmark historical evidence was cited for contextualization in the Discussion. In accordance with scoping review methodology, formal critical appraisal of included studies was not performed, allowing for the potential inclusion of studies with variable methodological quality. Furthermore, the frequent presence of concurrent chemical co-exposures (such as xylene or anesthetic gases) introduces confounding factors that are difficult to fully isolate when assessing formaldehyde-specific health outcomes. Finally, a major gap identified in the current literature is the near-total absence of research on non-neoplastic systemic effects of formaldehyde exposure in healthcare professionals, particularly regarding reproductive function, pregnancy, lactation, and neurological health.

Regarding the qualitative component, the study relied on a small purposive sample (14 participants) within a specific regional and institutional context. Although diverse professional profiles were included, the sample may not fully represent the broader healthcare workforce, thereby limiting the direct transferability of findings to other institutional or geographical settings. Procedural aspects may also have introduced response biases: distributing the interview guide in advance via email may have reduced response spontaneity and facilitated prepared or socially desirable answers. Similarly, providing preliminary background information on formaldehyde prior to focus groups, combined with certain structured questions acting as leading prompts, may have exerted a moderator priming effect on baseline risk perception accounts. Furthermore, time constraints prevented the conduct of a formal member-checking process for participant validation. Finally, observations concerning professional attitudes, productivity pressures, and safety culture should be interpreted as exploratory, context-dependent qualitative insights rather than universally generalizable conclusions.

## 7 Conclusion

This study demonstrates that occupational exposure to formaldehyde (FA) in healthcare settings represents a complex safety challenge where documented chemical and genotoxic risks intersect with substantial behavioral and institutional barriers. While current evidence confirms FA’s carcinogenicity and low-dose genotoxicity, hospital pathology and autopsy environments continue to exhibit recurring exceedances of exposure limits. Crucially, our findings reveal a profound disconnect between technical risk data and real-world clinical practice, characterized by a lower awareness of chemical hazards compared to biological risks, marked disparities in risk perception between physicians and non-physician staff, and institutional productivity pressures that hinder safety compliance.

Practically, addressing these challenges requires moving beyond formal regulatory frameworks toward active risk governance. Health organizations must prioritize engineering controls, such as localized table-level backdraft exhaust ventilation, and enforce standardized, job-specific training programs that foster a peer-supported safety culture. Furthermore, occupational health protocols must establish immediate, uniform protection for workers of childbearing age or who are pregnant.

From a research perspective, future studies must bridge current evidence gaps by conducting prospective, multicenter cohort studies specifically focused on healthcare personnel, incorporating individual personal dosimetry and longitudinal biomonitoring. Research should also expand beyond oncological endpoints to systematically evaluate non-neoplastic systemic effects, including reproductive toxicity and neurodegenerative markers. Ultimately, safeguarding healthcare workers requires an integrated approach that pairs updated, evidence-based regulatory limits with tailored educational and organizational interventions that transform risk perception into sustained preventive behavior.

## Data Availability

The original contributions presented in the study are included in the article/Supplementary material, further inquiries can be directed to the corresponding authors.
