# Supplementary material for: Chemical risk perception among healthcare workers: formaldehyde exposure as a model for occupational prevention challenges
Source: Front Public Health. 2026 Oct 22;14:1944564. doi: 10.3389/fpubh.2026.1944564 (PMC13736767; doi:10.3389/fpubh.2026.1944564)
Supplement: Supplementary file 1 [file Data_Sheet_1.docx]

Supplementary Material

# 1 Supplementary Figures and Tables

## 1.1 Supplementary Figures

**Supplementary Figure 1.** PRISMA 2020 flow diagram for new systematic reviews which included searches of databases, registers and other sources
