# Supplementary material for: Chemical risk perception among healthcare workers: formaldehyde exposure as a model for occupational prevention challenges
Source: Front Public Health. 2026 Oct 22;14:1944564. doi: 10.3389/fpubh.2026.1944564 (PMC13736767; doi:10.3389/fpubh.2026.1944564)
Supplement: Supplementary file 2 [file Supplementary_file_1.docx]

Appendix 1: Focus Group Participant Characteristics

Generated using NVivo software, where the area indicates the relative number of participants within each profile.
