# Supplementary material for: Chemical risk perception among healthcare workers: formaldehyde exposure as a model for occupational prevention challenges
Source: Front Public Health. 2026 Oct 22;14:1944564. doi: 10.3389/fpubh.2026.1944564 (PMC13736767; doi:10.3389/fpubh.2026.1944564)
Supplement: Supplementary file 3 [file Supplementary_file_2.docx]

Appendix 2: Focus Group Interview Guide

**FOCUS GROUP** 2025

**Moderator:** Lara García Asún

**Duration:** 2h30

**Language**: Spanish

**Objectives:**

- To explore the perceptions and attitudes of healthcare professionals toward chemical risk, using formaldehyde as a case example.
- To assess their level of knowledge and appraisal of chemical risk, and of personal and collective protective measures.
- To compare perceptions and attitudes toward chemical risk with those toward other occupational hazards (biological, psychosocial, physical, etc.), and across different healthcare professional profiles.
- To analyze training needs in occupational risk prevention from the undergraduate stage onward, and to identify potential gaps.

**WELCOME**

*Introduction by the moderator and assistant:*

Good afternoon, and welcome. Thank you for taking the time to participate in this session. Your perspectives are invaluable to our research, which seeks to understand how healthcare professionals from different specialties perceive their training in health, safety, and occupational risk prevention, both during their academic education and in professional practice.

My name is Lara García Asún. I will be accompanied by my supervisor MD-PhD Begoña Martínez Jarreta, who will take notes and provide support throughout the discussion.

Before we begin, you will be asked to complete a brief form collecting basic sociodemographic information, such as your specialty and years of experience, as well as an informed consent form for participation in this session. All information provided will be treated as strictly confidential and used exclusively for research purposes.

**INTERVIEW GUIDE AND OPENING QUESTIONS**

**1. Introduction by the moderator:**

- **Definition of Safety:** Although the concept of safety may vary across fields and contexts, it is commonly understood as the absence of risk.
- **Definition of Occupational Risk Prevention:** The discipline aimed at promoting the improvement of worker health and safety through the application of measures and the development of activities designed to prevent risks arising from working conditions.
- **Overview of Formaldehyde:** Formaldehyde is a chemical compound used primarily in the manufacture of resins and industrial products. In healthcare settings, it is used as a biological tissue fixative in anatomical pathology, as a component of formalin, and is also present in certain cleaning products used by hospital staff. Exposure to formaldehyde can occur in various hospital areas, not only in laboratories, but also in outpatient clinics, operating theatres, and endoscopy units.

In the short term, inhalation of or skin contact with formaldehyde can cause irritation of the eyes, respiratory tract, and skin, with symptoms including eye irritation, cough, and difficulty breathing. In the long term, it is associated with an increased risk of cancer, particularly nasopharyngeal cancer, as well as other genotoxic effects affecting DNA. For this reason, the International Agency for Research on Cancer (IARC) classifies it as a Group 1 carcinogen.

Continuous exposure to this chemical agent raises significant concerns regarding the health of healthcare workers, underscoring the need for adequate protective measures and occupational safety training.

- **Objectives:**
  - To explore physicians' perceptions and attitudes toward chemical risk, using formaldehyde as a case example.
  - To assess their level of knowledge and appraisal of chemical risk, and of personal and collective protective measures.
  - To compare perceptions and attitudes toward chemical risk with those toward other occupational hazards (biological, psychosocial, physical, etc.), and with those of other healthcare professionals.
  - To analyze training needs in occupational risk prevention from the undergraduate stage onward, and to identify potential gaps.

**2. Guiding considerations:**

- Discussion will proceed from the general to the specific.
- We are interested in exploring whether participants have received training in the following areas:
  - Health
  - Safety
  - Risk prevention
  - Occupational risk prevention

**3.** The audio recorder will be activated with the participants' consent.

**4. Discussion Questions:**

**Definition of Chemical Risk (Formaldehyde as a case example)**

- Were you previously aware of the forms and risks of formaldehyde exposure?
- In what situations are you exposed to formaldehyde in your workplace?

**Perception of Chemical Risk**

- Do you consider formaldehyde to represent a significant health risk in your occupational environment?
- Are you aware of other carcinogens to which healthcare workers may be exposed?
- On a scale of 1 to 5, how would you rate the importance of the following occupational risks: chemical (formaldehyde), biological (infections), psychosocial (stress, burnout), and physical (radiation, ergonomics)? Where 1 indicates 'minimal perceived risk/concern' and 5 indicates 'maximum perceived risk/concern'.
- Do you consider the management of chemical risks to be more or less effective than that of other occupational hazards?

**Personal and Collective Protective Measures**

- What protective measures are you aware of and do you use in relation to formaldehyde and other chemical risks in your workplace? Do you consider them adequate and sufficient?
- What gaps or unmet needs do you perceive with regard to protection against these risks?

**Occupational Risk Prevention Training**

- What occupational risk prevention training did you receive during your undergraduate studies?
- Do you feel you should have received more training on chemical risk protection during your education?
- Have you received any occupational risk prevention training or updates throughout your professional career?

**Regulatory Framework**

- Are you familiar with the concept of criminal liability for offenses against worker health?
- Were you aware that diseases caused by formaldehyde exposure are classified as occupational diseases?

**General Discussion**

- Do you perceive psychological pressure among healthcare workers to prioritize professional vocation and patient care over their own personal health protection?
