# Supplementary material for: Chemical risk perception among healthcare workers: formaldehyde exposure as a model for occupational prevention challenges
Source: Front Public Health. 2026 Oct 22;14:1944564. doi: 10.3389/fpubh.2026.1944564 (PMC13736767; doi:10.3389/fpubh.2026.1944564)
Supplement: Supplementary file 4 [file Supplementary_file_3.docx]

Appendix 3: Data Analysis Process Using NVivo Software

Summary of core qualitative themes identified during focus group sessions on occupational formaldehyde risk perception among healthcare professionals.

Overarching thematic classification mapping the primary domains of formaldehyde risk perception and preventive behaviors.

Detailed breakdown of specific qualitative subthemes mapped across each core thematic domain.
