# Supplementary material for: Chemical risk perception among healthcare workers: formaldehyde exposure as a model for occupational prevention challenges
Source: Front Public Health. 2026 Oct 22;14:1944564. doi: 10.3389/fpubh.2026.1944564 (PMC13736767; doi:10.3389/fpubh.2026.1944564)
Supplement: Supplementary file 5 [file Supplementary_file_4.docx]

Appendix 4: Results table

Chronic effects

| Reference | Year | Document type | Country/Region | Population studied | Source | Health effects |
| --- | --- | --- | --- | --- | --- | --- |
| Kwon et al. | 2018 | Systematic review | South Korea | 54,000 workers in pathological anatomy laboratories, endoscopy facilities, and furniture manufacturing. | Database | This study demonstrates a significant association between formaldehyde exposure and the development of leukemia (particularly myeloid leukemia) and Hodgkin’s lymphoma, with elevated risks at peak exposures of ≥4 ppm (RR 2.47 for myeloid leukemia). However, the relationship with nasopharyngeal cancer (NPC) is less clear, showing significant results in case-control studies but not in cohort studies, suggesting the influence of environmental factors or biases. Although South Korea recognized formaldehyde as an occupational carcinogen in 2013, no cases have been compensated solely for this exposure. |
| Ghelli et al. | 2021 | Case-control study | Italy | Exposed group: 23 hospital workers (nurses, pathology technicians, and pathologists) Control group: 45 unexposed hospital workers | Database | Workers exposed to formaldehyde showed significantly higher levels of oxidative stress (15-F₂ₜ-isoprostane and MDA) and inflammation (TNF-α) compared to unexposed workers (p < 0.001).  A positive correlation was observed between airborne formaldehyde concentration and biomarkers of oxidative stress (MDA: rho = 0.647, p < 0.001).  Subjects with GSTT1 and GSTM1 genotypes and carriers of mutated alleles in CYP1A1 had higher MDA levels (p = 0.038, 0.031, and 0.008, respectively), suggesting greater vulnerability to oxidative damage. |
| Ghelli et al. | 2022 | Case-control study | Italy | Exposed group: 57 pathologists  Control group: 48 unexposed healthcare workers | Database | Pathologists exposed to formaldehyde showed a significantly higher frequency of sister chromatid exchanges (SCEs) in blood lymphocytes, confirming its genotoxic effect even at low concentrations. The concentration of formaldehyde in the air correlated directly with SCEs and inversely with the replication index (RI), indicating cytotoxicity.  Although some polymorphisms (CYP1A1 exon 7 and XPC exon 9) showed an association with a higher frequency of SCEs in exposed individuals, regression models did not reveal a significant role for the analyzed genetic variants in modulating genotoxic damage. |
| Ghelli et al. | 2021 | Case-control study | Italy | Exposed group: 57 pathologists  Control group: 48 unexposed hospital workers | Database | The study confirmed that pathologists exposed to occupational formaldehyde (FA) had significantly higher rates of chromosomal aberrations (CAs) compared to controls (p < 0.001), demonstrating its genotoxic effect even at low concentrations (mean: 64.2 µg/m³). Furthermore, it identified that polymorphisms in metabolism genes (CYP1A1 exon 7 and CYP2C19*2) increased susceptibility to genetic damage, while variants in GSTT1 and GSTM1 exerted a protective effect. |
| Akhlaghi et al. | 2023 | Cohort study | Iran | Exposed group: 64 pathologists  Control group: 32 unexposed administrative staff | Database | The study demonstrated that formaldehyde exposure among workers in pathological anatomy laboratories is associated with a significant increase in the frequency of micronuclei (MN) in oral mucosal cells (18.13% vs. 10.55% in controls, p=0.003), indicating genotoxic damage. Although no significant relationship was found between years of exposure and the frequency of MN (p=0.17), daily exposure time showed a direct and significant correlation (p=0.03). There were no differences by gender or age. |
| Jalali et al. | 2021 | Cross-sectional descriptive study | Iran | 60 pathology laboratory employees | Database | The study revealed that pathology laboratory employees are exposed to mean formaldehyde levels (0.64 mg/m³) exceeding the limits recommended by NIOSH and national standards, with 28.3% of cases exceeding the OSHA limit. The mean carcinogenic risk (3.45 × 10⁻⁴) was unacceptable for 81.7% of workers according to occupational standards, while non-carcinogenic risk varied depending on the reference benchmark (HQ = 178.49 for the general population vs. 0.69 for workers). |
| International Agency for Research on Cancer (IARC) | 2012 | Scientific Report (Monograph) | Internacional |  | Grey literature | The International Agency for Research on Cancer (IARC) Monograph 100F, published in 2012, evaluates the carcinogenicity of formaldehyde based on international studies conducted in countries such as the United States, China, and various regions of Europe. This scientific document concludes that there is sufficient evidence to state that formaldehyde is a human carcinogen (Group 1), primarily associated with nasopharyngeal cancer and leukemia, while data on its link to paranasal sinus cancer, although positive, are less conclusive. In animal studies, its ability to induce nasal tumors in rats and mice was demonstrated, supporting its carcinogenic potential. Proposed mechanisms include genotoxicity, such as DNA damage and adduct formation, as well as cytotoxic effects on exposed tissues. Occupational exposure in industries such as furniture manufacturing, textiles, and funeral or medical services poses a high risk, while environmental sources such as tobacco smoke, building materials, and vehicle emissions also contribute to exposure. These findings confirm formaldehyde as a high-risk compound for human health. |

Regulations and Strategic Framework

| Author/Organization | Year | Document type | Country/Region | Source | Policy/Strategy |
| --- | --- | --- | --- | --- | --- |
| European Commission (Employment, Social Affairs and Inclusion) | 2021 | Official Statement | European Union | Grey literature | The European Commission (2021) presented a strategic framework for occupational safety and health (2021–2027) in the EU, focused on three key objectives:  (1) managing the changes resulting from the green, digital, and demographic transitions  (2) improving the prevention of workplace accidents and occupational diseases (e.g., cancer, which accounts for 52% of work-related deaths)  (3) increasing preparedness for health crises.  Notable data include a 70% reduction in fatal accidents (1994–2018), yet there are still 3,300 deaths annually and costs amounting to 3.3% of GDP (€460 billion). Proposed actions include updating legislation, addressing psychosocial risks, and protecting vulnerable groups, including healthcare workers. The framework promotes international cooperation (ILO/WHO) and funding through EU funds (ESF+, Horizon Europe). |
| Occupational Safety and Health Administration (OSHA), U.S. Department of Labor. | 2021 | Regulation/Legal Standard (Occupational Safety and Health Regulations) | USA | Grey literature | This document establishes occupational safety and health standards for formaldehyde exposure in the workplace, as regulated by the U.S. Occupational Safety and Health Administration (OSHA). It includes key definitions, such as the Permissible Exposure Limit (PEL) of 0.75 ppm as a time-weighted average (TWA) and a Short-Term Exposure Limit (STEL) of 2 ppm over 15 minutes. It also details requirements for exposure monitoring, engineering controls, personal protective equipment, medical surveillance, risk communication, and specific employee training. In addition, it provides technical information on formaldehyde, its health effects, emergency procedures, and analytical methods for measuring its concentration in the air. The document also addresses specific situations, such as regulated areas, emergency management, and recordkeeping, with the aim of protecting workers from the risks associated with formaldehyde exposure. |
| Loomis et al. | 2018 | Review | France | Organiztion | The study updates the list of occupational carcinogens identified by the IARC Monographs between 1971 and 2017, using strict criteria to ensure occupational relevance and strong causal evidence. Forty-seven occupational carcinogens (substances, mixtures, or radiation) were identified and classified in IARC Group 1 as having “sufficient evidence of carcinogenicity” in humans, based on studies of exposed workers. The most frequently associated cancers were lung (23%), skin (10%), and bladder (7%). The main routes of exposure were inhalation and dermal contact. The document highlights the need for further research to assess carcinogenic risks in occupational settings, especially in low- and middle-income countries, and notes methodological advances such as improvements in the quantitative assessment of exposures. The list represents a conservative estimate due to the lack of epidemiological data for many occupational exposures. |
| International Labour Organization (ILO) | 2022 | Official conference document | International | Grey literature | The 110th International Labour Conference (ILC), organized by the International Labour Organization (ILO) in 2022, recognized occupational safety and health (OSH) as a fundamental right, adopting key resolutions to strengthen the protection of workers against occupational hazards, including exposure to carcinogens such as formaldehyde. This official document, with global reach and endorsed by the ILO’s 187 member states, emphasizes the need to harmonize national regulations with international standards, such as Convention 155, and highlights the importance of implementing specific preventive measures for high-risk sectors, such as healthcare. The resolution underscores the urgency of developing rigorous protocols to monitor and reduce exposure to hazardous substances, providing a framework for assessing compliance with Spanish legislation (e.g., Law 31/1995 on Occupational Risk Prevention) with ILO guidelines. |
| European Commission (Directorate-General for the Environment and Directorate-General for Growth) in collaboration with the European Chemicals Agency (ECHA) | 2022 | Policy Implementation Guide | European Union | Grey literature | The European Commission’s working document (SWD/2022/128), published in April 2022 as part of the EU Chemicals Sustainability Strategy, establishes a “Rolling List” to prioritize the restriction of hazardous substances under the REACH Regulation. Among the identified groups, Pool 2 includes formaldehyde (classified as a Group 1B carcinogen) due to its occupational risk in healthcare sectors, such as pathological anatomy and hospital disinfection, where current exposure limits may be insufficient. The document proposes assessing the need for a specific restriction to protect workers, which implies potential updates to EU and Spanish regulations. |
| Instituto Nacional de la Seguridad y salud en el Trabajo (INSST) | 2024 | Regulatory Reference Manual | Spain | Grey literature | This document sets out the occupational exposure limits that Directive 98/24/EC imposes on Member States. Formaldehyde was added to this guide in 2018.  It is noted that it is not possible to identify exposure levels below which there is no risk that mutagens and carcinogens will have adverse health effects. An exposure-risk relationship is acknowledged, suggesting that the lower the exposure to these agents, the lower the risk.  For formaldehyde:  ELV-DE (environmental limit value—daily exposure): 0.3 ppm - 0.37 mg/m³  ELV-SE (environmental limit value—short-term exposure): 0.6 ppm - 0.74 mg/m³ |
| Ministerio de Trabajo y Asuntos Sociales (Spain) | 2006 | Royal Decree | Spain | Grey literature | This Royal Decree approves the list of occupational diseases, specifying the high-risk occupational activities—including those performed by healthcare personnel—within the Social Security system, and establishes criteria for their reporting and registration.  Diseases associated with exposure to formaldehyde are classified under the group of occupational diseases caused by chemical agents (Group 1), as well as under the group of occupational skin diseases caused by substances and agents not included in any of the other sections (Group 5). This means that diseases caused by formaldehyde suffered by a worker while performing the activities described in the current table may be classified as occupational diseases if a causal link is demonstrated. |

Prevention and Protection

| Reference | Year | Document type | Country / Region | Relevant population | Source | Evaluated/recommended strategies |
| --- | --- | --- | --- | --- | --- | --- |
| Charlier et al | 2021 | Narrative review | Italy | Healthcare workers, other hospital staff, and patients | Database | The article highlights the importance of environmental monitoring, biomonitoring, and predictive modeling strategies for estimating the risks of long-term formaldehyde exposure. It is based on the exposure limits established by OSHA and the WHO.  The prevention measures highlighted include substitution, local exhaust ventilation systems, personal protective equipment (PPE), and risk prevention training. Current challenges include regulatory harmonization and the need for a “One Health” approach. |
| Yee et al. | 2022 | Systematic review | Malaysia | Pathology Laboratories | Database | This review compares the evidence on various natural fixatives as alternatives to formaldehyde based on their fixing properties and staining quality.  Honey, jaggery, and sugar are evaluated. Honey shows promising results, but it is noted that the process and concentrations would need to be optimized before it can be put into practice.  30% of the jaggery fixatives showed better staining than formalin, but the studies are inconsistent. |
| Mucci et al. | 2019 | Analytical observational study | Italy | Healthcare workers | Database | The study concludes that the implementation of an automatic real-time sampling system proved superior to traditional methods for detecting intermittent spikes in formaldehyde exposure, particularly in areas such as pathology and morgues.  Personal dosimeters were more accurate than fixed environmental samplers in assessing actual individual exposure among staff.  The combination of forced ventilation, substitution with less toxic alternatives, and strict use of personal protective equipment significantly reduced exposure.  The study underscores the need to update monitoring protocols, train professionals in risk prevention, and adapt control measures to specific tasks. |
| Yahyaei et al | 2020 | Observational cross-sectional study | Iran | 62 employees in pathology departments (8 pathologists, 10 laboratory technicians, 7 administrative staff, 32 cleaning staff, and 5 laboratory workers) | Database | The study revealed that workers in the pathology departments of the five hospitals evaluated were exposed to formaldehyde concentrations (0.0192–0.326 ppm) that exceeded the NIOSH-recommended limit (0.016 ppm), although they remained below the OSHA standard (0.75 ppm), except in one hospital. The calculated cancer risk (LCP) ranged from 9.52×10⁻⁵ to 1.53×10⁻³, significantly exceeding the WHO acceptable level (10⁻⁶ to 10⁻⁵), with laboratory technicians being the most vulnerable group. The main factors associated with high exposure included inadequate ventilation, long working hours, and small room sizes. These findings highlight the need to implement control measures, such as improving ventilation systems, optimizing exposure times, and ensuring the proper use of personal protective equipment. |
| Foroughi et al. | 2024 | Observational cross-sectional study | Iran | 57 employees of pathological anatomy laboratories (pathologists, laboratory technicians, cleaning staff) | Database | The study revealed that 91.23% of workers in pathology laboratories were exposed to formaldehyde levels (0.0192–0.326 ppm) that exceeded the NIOSH-recommended limit (0.016 ppm). Laboratory technicians and pathologists had the highest exposure, with 41.03% of cases at definite carcinogenic risk (LCR > 10⁻⁴) and 76.92% at unacceptable non-carcinogenic risk (HQ > 1). In addition, a high prevalence of respiratory symptoms was observed, such as dyspnea (80%), secretions (70%), and cough (58.39%). The results call for measures such as improved ventilation, reduced exposure time, use of personal protective equipment, and training. |
| Fustinoni et al. | 2021 | Longitudinal intervention study | Italy | 29 pathology laboratory employees (technicians, pathologists, residents, cleaning staff) | Database | Critical exposures (>0.37 mg/m³) were detected among pathologists and residents during macroscopic examination, exceeding acceptable occupational limits. Interventions that proved effective in reducing exposure included spatial reorganization (closed fume hoods), reduced use of formalin, and risk prevention training. As a result, personal and environmental exposures to formaldehyde were reduced, meeting current occupational exposure limits by 2020. |
| Wang et al. | 2023 | Intervention study | China | Exposure group: 22 pathologists  Control group: 24 medical professionals who are not pathologists | Database | The study showed that comprehensive interventions (such as reducing the amount and frequency of formaldehyde use, upgrading equipment, and training staff) lowered formaldehyde concentrations in pathology departments, thereby reducing health risks to staff. However, a moderate carcinogenic risk remains, particularly in technical rooms. |
| Kangarlou et al. | 2024 | Observational cross-sectional study | Iran | 133 hospital laboratory staff members (74 workplaces) | Database | The study revealed that, although average formaldehyde exposure levels were below occupational limits (OSHA PEL: 0.75 ppm), 20% of workers in high-risk departments (pathology and infectious diseases) exceeded these limits, highlighting the need for individualized monitoring. The risk assessment showed that the risk of cancer (ELCR) and non-cancer effects (HQ) remained within safe thresholds, but a high prevalence of respiratory symptoms (coughing, wheezing) and irritation (ocular, pharyngeal) was recorded, associated with areas of higher exposure. The findings support the implementation of control measures such as localized ventilation, use of PPE, and adequate training, as well as the adoption of an integrated approach (environmental sampling, biomarkers, and health questionnaires) for a more accurate risk assessment in work environments with formaldehyde. |
| Motta et al. | 2021 | Longitudinal observational study | Italy | 16 healthcare workers exposed to formaldehyde in a Pathology Department | Database | The study results showed that ambient formaldehyde (FA) levels in the Pathology Unit were low on average, although some spot measurements exceeded the limits set by NIOSH. Urinary FA levels among workers were minimal and remained well below the reference value (5.6 mg/L), although a slight weekly accumulation was observed. In addition, a significant improvement in compliance with the limits was observed over time, attributable to increased awareness and improved practices among workers. |
| Protano et al. | 2024 | Systematic review | Italy | Adult workers occupationally exposed to formaldehyde in healthcare, research, and industrial settings | Database | The systematic review showed that, while biomarkers of formaldehyde exposure (such as formic acid in urine) have limitations due to their low specificity and individual variability, biomarkers of early effects, particularly cytogenetic assays (micronuclei in lymphocytes and buccal cells), are sensitive tools for assessing genotoxic damage in occupationally exposed workers. The included studies showed significant increases in these biomarkers, supporting their utility in occupational risk surveillance. However, the influence of factors such as diet, concurrent exposure to other carcinogens, and individual differences underscores the need to adjust the analyses for these confounding factors |
| Ramírez Pérez et al. | 2018 | Scoping review | Spain | Workers exposed to formaldehyde | Organization | Exposure levels to formaldehyde (FA) and their regulation vary significantly depending on the workplace and geographic context. In Spain, the INSHT (2018) sets environmental limit values of 0.3 ppm for daily exposure (VLA-ED) and 0.6 ppm for short-term exposure (VLA-EC), while international organizations such as OSHA and NIOSH in the U.S. propose less restrictive limits (0.75 ppm and 0.016 ppm, respectively). Measurements in hospital settings, such as the pathology department, show wide ranges (0.08–6.90 ppm), with peaks of up to 8.40 ppm in autopsy rooms. Regarding biomarkers, formic acid in urine has limitations due to its low specificity and individual variability, which has led to conflicting results in studies. Therefore, it is recommended to supplement its analysis with tests such as spirometry, complete blood counts, and genotoxicity assessments (micronuclei in lymphocytes), integrating these parameters into medical surveillance protocols tailored to the specific risks of each sector. These measures are urgent given the classification of FA as a human carcinogen (IARC Group 1) and the evidence of its chronic effects, although gaps remain in research on its reproductive impacts and in the validation of more reliable biomarkers, which calls for more robust epidemiological studies and alternatives to the use of FA in high-risk settings. The article highlights the importance of conducting a comprehensive clinical and occupational history in workers exposed to formaldehyde (FA) as a fundamental part of their medical follow-up. |
| Sociedad Española de Anatomía Patológica (SEAP-IAP) | 2016 | Technical Recommendations Document and Best Practices Guide. | Spain | Pathology laboratory workers | Grey literature | This document provides guidelines for the safe handling of formaldehyde and the use of alternative fixatives in the healthcare setting. Formaldehyde, reclassified as a Group 1 carcinogen by the IARC and 1B carcinogen and 2 mutagen by the EU CLP (Regulation 605/2014), poses health risks, such as acute irritation and chronic effects (asthma, cancer), with a permissible exposure limit of 0.3 ppm. To minimize these risks, the document recommends structural measures (closed supply systems, carving booths with air extraction and specific filters) and the use of personal protective equipment (PPE) such as masks with ABEK or B2P3 filters, sealed goggles, and nitrile gloves. In addition, alternative fixatives based on glyoxal or propylene glycol are proposed; although flammable, they are less toxic and offer better preservation of DNA/RNA for molecular studies, though they require adjustments to techniques such as immunohistochemistry. The document emphasizes the need to validate these fixatives for specific diagnostics and recommends their gradual adoption, while continuing to use formaldehyde for tumor cases until international standards are established. It also highlights the importance of training, multidisciplinary risk assessment, and compliance with regulations such as Regulation 605/2014 and Royal Decree 665/1997. |
| Centers for Disease Control and Prevention (CDC) | 2019 | Technical Guide | USA | Workers exposed to formaldehyde | Grey literature | The guide establishes the following exposure limits: NIOSH  Recommended Exposure Limit (REL)  - Time-Weighted Average (TWA): 0.016 ppm  - Short-Term Exposure Limit (STEL): 0.1 ppm (15 minutes)  OSHA  Permissible Exposure Limit (PEL)  - TWA: 0.75 ppm  - STEL: 2 ppm (15 minutes)  The Immediately Dangerous to Life or Health (IDLH) level is set at 20 ppm, based on its classification as an occupational carcinogen (linked to nasal cancer).  Regarding protective measures, the document specifies:  - Respiratory protection: For concentrations above the REL, it requires self-contained positive-pressure respirators (APF 10,000) or full-facepiece air-supplied respirators.  - Skin and eye protection: It indicates the need to avoid all contact by wearing gloves and protective suits  Regarding measurement methods, it complies with NIOSH (2016, 2541, 3500, 3800) and OSHA (ID205, 52) standards for environmental monitoring. |
| Yale University, Office of Environmental Health & Safety (EHS) | 2024 | Institutional Policy Document | USA | Workers/students exposed to formaldehyde | Grey literature | A regulatory document establishing protocols for the safe handling of formaldehyde (≥0.1%), in accordance with OSHA standard 29 CFR 1910.1048. It specifies exposure limits (0.75 ppm PEL, 2 ppm STEL) and requirements such as biannual environmental monitoring (NIOSH/OSHA methods), engineering controls (fume hoods with a flow rate of ≥100 fpm), and PPE (nitrile gloves, APF 10,000 respirators if the STEL is exceeded). It includes annual medical surveillance for those exposed to ≥0.5 ppm, with records retained for 30 years. The program covers high-risk activities (anatomy, pathology) and emphasizes the importance of annual training, hazardous waste disposal, and appendices with SDSs and guidelines for anatomical dissection, ensuring legal compliance and occupational safety in academic and clinical settings. |
| National Institute for Occupational Safety and Health (NIOSH) | 2022 | Informational white paper | USA | Pregnant/breastfeeding workers | Grey literature | This document warns of the reproductive risks associated with occupational exposure to formaldehyde, including potential fertility issues and an increased risk of miscarriage, particularly among workers in funeral homes, anatomy/pathology laboratories, and beauty salons. It notes that, although most exposures do not reach significant levels in breast milk, concentrations above 2 ppm—such as those found in some laboratories—could be transferred during breastfeeding. The text details exposure limits (OSHA PEL: 0.75 ppm TWA, 2 ppm STEL; NIOSH REL: 0.016 ppm TWA) and recommends protective measures such as the use of nitrile/butyl gloves, adequate ventilation, post-exposure washing, and temporary relocation for pregnant women, noting that current standards do not fully account for fetal risks. It includes legal resources (Pregnant Workers Fairness Act) and emphasizes medical consultation to assess individual risks, providing specific protocols to reduce exposure in high-risk environments. |
| Instituto Nacional de Seguridad y Salud en el Trabajo (INSST) | 2021 | Technical Data Sheet (Occupational Safety and Health Guide) | Spain | Workers exposed to formaldehyde | Grey literature | This fact sheet describes formaldehyde as a colorless, flammable, and highly reactive gas used in resins, disinfectants, textiles, wood panels, and the healthcare (formalin) and food industries. It is also present in vehicle emissions, tobacco smoke, and household products. Its health effects include acute irritation of the respiratory tract, skin, and eyes; lethal toxicity at high doses; and chronic effects such as asthma, dermatitis, and skin sensitization. Furthermore, it is classified as a carcinogen (IARC Group 1) associated with myeloid leukemia and nasopharyngeal cancer. In the EU, its use is regulated (Carc. 1B, Mutagen Cat. 2) with restrictions on cosmetics and toys. The sectors with the highest occupational exposure are furniture manufacturing (179,000 workers) and medical services (174,000), with a limit value of 0.3 ppm (VLA-ED®). Preventive measures prioritize substitution, closed systems, and PPE (BP filters). Health surveillance includes protocols for dermatitis and asthma, with post-exposure monitoring. Key references include Royal Decree 665/1997 and Regulation (EC) No. 1272/2008 (CLP). |
